# Floral Niche Selection by a Generalist Predator: Chemo-Orientation of *Orius maxidentex* to *Celosia argentea* Volatiles

**DOI:** 10.3390/biology15080658

**Published:** 2026-04-21

**Authors:** Yinyi Liu, Wei Gan, Xia Shi, Zhengpei Ye, Fan Song, Hu Li, Wanzhi Cai, Jianyun Wang, Junyu Chen

**Affiliations:** 1 Environment and Plant Protection Institute, Chinese Academy of Tropical Agricultural Sciences, Haikou 570100, China; liuyinyi@cau.edu.cn (Y.L.); ganwei@catas.cn (W.G.); xiashi936@sina.com (X.S.); zhengpeiyecn@163.com (Z.Y.); 2 State Key Laboratory of Agricultural and Forestry Biosecurity, MOA Key Lab of Pest Monitoring and Green Management, Department of Entomology, College of Plant Protection, China Agricultural University, Beijing 100193, China; fansong@cau.edu.cn (F.S.); tigerleecau@hotmail.com (H.L.); caiwz@cau.edu.cn (W.C.); 3 Hainan Provincial Engineering Research Center for the Breeding and Industrialization of Natural Enemies, Haikou 571101, China

**Keywords:** behavioral responses, *Celosia argentea*, insect–plant interactions, *Orius maxidentex*, volatile organic compounds

## Abstract

Plant volatiles are critical mediators of insect–plant interactions, guiding natural enemies to specific habitats and prey. The flower bug, *Orius maxidentex* Ghauri (Hemiptera: Anthocoridae), is a generalist predator that exhibits a specialized ecological association with the weed *Celosia argentea* L. (Caryophyllales: Amaranthaceae), utilizing the plant as a primary floral niche in Hainan Island. In this study, the attractiveness of *C. argentea* floral volatiles to *O. maxidentex* was confirmed using a Y-tube olfactometer. Solid-phase microextraction (SPME) combined with gas chromatography–mass spectrometry (GC-MS) was utilized to identify six compounds in the floral volatiles: 1,3-diethenylbenzene, trans-cinnamaldehyde, β-bisabolene, methyl salicylate, 3-ethylbenzaldehyde, and nonanal. Electroantennogram (EAG) assays revealed that *O. maxidentex* antennae showed significant physiological responses to these compounds, and the EAG relative values were positively correlated with concentration gradients. Furthermore, *O. maxidentex* exhibited significant orientation responses to 1,3-diethenylbenzene, trans-cinnamaldehyde, β-bisabolene, and methyl salicylate, whereas no behavioral response was observed for 3-ethylbenzaldehyde or nonanal. Further tests revealed that β-bisabolene elicited the highest attractiveness, comparable to a synthetic blend formulated to mimic the natural release ratio of the active semiochemicals. These findings reveal the hidden chemical cues mediating the interaction between a predator and its preferred habitat. Understanding this mechanism not only helps explain insect adaptation but also offers new strategies for using these plant volatiles to influence the behavior of this specific predator, potentially enhancing its targeted recruitment in agroecosystems.

## Simple Summary

Predatory insects are essential for managing agricultural pests, but they also rely on plants for shelter and supplementary food, such as pollen and nectar. The flower bug, *Orius maxidentex* Ghauri (Hemiptera: Anthocoridae), is a generalist predator that plays a crucial role in tropical ecosystems. While it is considered to be a promising biological control agent preying on small-sized pests, it shows a strong preference for inhabiting the inflorescences of a specific weed *Celosia argentea* L. (Caryophyllales: Amaranthaceae), as a primary habitat. However, the specific chemical cues that guide the flower bug to its host plant remain unknown. In this study, we collected the floral scents of *C. argentea* and tested how the flower bugs reacted to them using both electrophysiological sensors on their antennae and behavioral choice tests. We found that the bugs are highly sensitive to six specific compounds released by the flowers. Among them, the compound β-bisabolene was identified as the most attractive semiochemical. This study identifies key attractants driving the recruitment of *O. maxidentex* to *C. argentea*, highlighting the specific behavioral responses of this predator to floral olfactory cues.

## 1. Introduction

Angiosperms and insects form the two most diverse groups of multicellular organisms on Earth [1]. Flowers, the reproductive organs of angiosperms, serve as key mediators of plant–insect interactions by emitting a variety of sensory signals and have developed intricate ecological relationships with insects through long-term reciprocal adaptations in physiological, biochemical, and morphological traits [2,3,4]. Flowers transmit signals to potential visitors through multiple channels, including petal color, petal shape, corolla morphology, and volatile emissions. The fundamental relationship between diverse chemical signals and behavior of arthropods occupies a prominent position in ecological research [5], and semiochemicals released by organisms into the surrounding environment are pivotal in maintaining or disturbing interspecific interactions [6,7]. It is well known that flower color and floral scent assist insects in locating host plants. In this process, visual cues such as color generally function only at close range, whereas floral scent can serve as a long-distance attractant [8,9]. Among these, the role of floral scent, a blend of volatile organic compounds (VOCs) produced and emitted by the different floral parts (e.g., petal, pollen, nectar) is fundamental. Insects, like most animals, rely heavily on their olfactory systems to discriminate ecologically relevant airborne cues or signals from random information, thereby making decisions that influence foraging, mating, nesting, and defending themselves against natural enemies [5,10,11].

**Figure 1:**
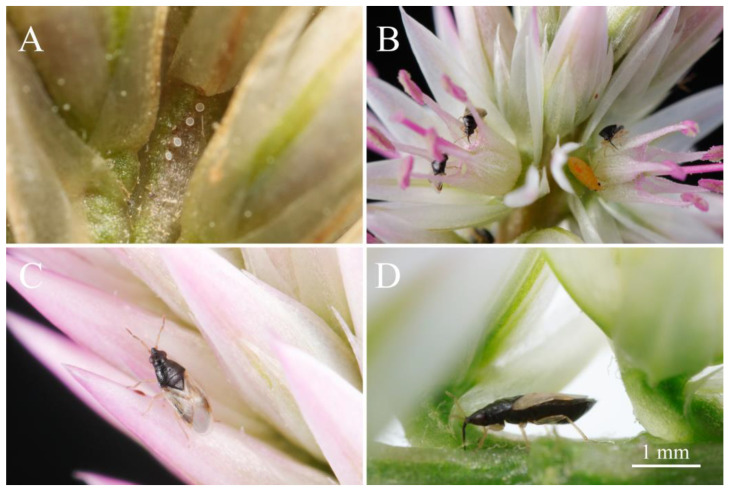
Habitats of *Orius maxidentex* Ghauri. (**A**) Eggs laid in the pedicel of *Celosia argentea* L.; (**B**) adults and nymphs aggregating on the inflorescence; (**C**) dorsal view of male adult; (**D**) gravid female adult feeding on plant sap.

For generalist predators, efficiently locating suitable micro-habitats is crucial not only for finding prey but also for fulfilling nutritional requirements (e.g., pollen and nectar) and identifying optimal oviposition sites [12]. Unlike specialists that track specific prey pheromones, generalist predators often rely on plant-derived volatiles to locate habitats that offer both prey and supplementary plant foods. The flower bug, *Orius maxidentex* Ghauri (Hemiptera: Anthocoridae), is an endemic predator in tropical regions and is considered a promising biological control agent preying on thrips, aphids, and other small-sized agricultural pests [13,14,15]. Our research team previously found that *O. maxidentex* gathered in large numbers on the inflorescences of *Celosia argentea* L. (Caryophyllales: Amaranthaceae) in Hainan Island, with both adults and nymphs occurring simultaneously on the same plant [14] (Figure 1). The gravid female individuals often lay eggs on the base of fresh flower stalks, and their populations track flowering *C. argentea* both spatially and temporally, even in the absence of prey. This suggests that *C. argentea* serves as a critical floral niche and habitat for this predator. Furthermore, we recently characterized the antennal sensilla of *O. maxidentex* and identified specific chemoreceptors likely responsible for olfactory perception, providing the morphological basis for their chemical communication [16]. However, the specific chemical composition of *C. argentea* volatiles and the mechanism by which *O. maxidentex* perceives and responds to these cues remain virtually unexplored.

While it is well established that herbivore-induced plant volatiles (HIPVs) recruit natural enemies to infested plants [17,18,19,20], the role of constitutive floral volatiles in recruiting generalist predators to uninfested plants remains poorly understood. *Orius* species are zoophytophagous, meaning they can complete their development on plant resources such as pollen [21,22,23]. Lorenzo et al. [24] reported that *Orius insidiosus* (Say) and *Orius tristicolor* (White) preferred the volatiles emitted by flowering strawberry plants in Y-tube experiments, which confirmed that females rely on chemical and olfactory cues to recognize suitable host plants. While certain ubiquitous floral volatiles, such as the sesquiterpene β-bisabolene, are known to act as semiochemicals in various ecological interactions, their specific role in recruiting generalist predators like *O. maxidentex* to a key habitat plant remains uncharacterized. Based on our field observations and morphological findings, we hypothesized that specific VOCs emitted by flowering *C. argentea* mediate the habitat selection and host preference of *O. maxidentex*, acting as primary attractants independent of prey-associated signals.

The aim of this study was to elucidate the chemosensory basis of the interaction between the flower bug *O. maxidentex* and the flowers of *C. argentea*. In this study, we investigated whether the predator utilizes floral volatiles of uninfested *C. argentea* as primary cues for habitat location and identified the key active compounds involved. To achieve this, we characterized the floral volatile profile of *C. argentea* using solid-phase microextraction (SPME) combined with gas chromatography–mass spectrometry (GC-MS); screened for physiologically active compounds using electroantennography (EAG); and evaluated the behavioral responses of *O. maxidentex* to individual compounds and blends in olfactometer bioassays. Our findings provide new insights into the olfactory adaptations of this tropical predator and the chemical mechanisms underlying its niche selection, which may contribute to a better understanding of predator–plant interactions in similar systems and offer insights for developing biological control strategies involving predatory insects in tropical agroecosystems.

## 2. Materials and Methods

### 2.1. Insects and Plants

*Orius maxidentex* were initially collected from *C. argentea* in the field of Chinese Academy of Tropical Agricultural Sciences in Danzhou, Hainan Province, China (19°30′ N, 109°29′ E), and used to establish a laboratory colony. In the laboratory, the *O. maxidentex* population was maintained in nylon mesh cages (50 × 50 × 50 cm) in a climate-controlled room under a temperature of 26 ± 1 °C, a relative humidity (RH) of 75 ± 5%, and a 16 L: 8 D photoperiod. Potted soybean seedlings, *Glycine max* (L.) Merr. (Fabales: Fabaceae), infested with the prey *Echinothrips americanus* Morgan (Thysanoptera: Thripidae) were placed inside the cages. These infested seedlings served simultaneously as a food source, a moisture provider, and an oviposition substrate for the predatory bugs. To ensure a continuous and sufficient prey supply, the prey-infested soybean seedlings were replaced with fresh ones every 7 days. For all electrophysiological and behavioral assays, newly emerged adult females were collected from the colony and starved for 3 h prior to testing.

The flowers of *C. argentea*, *Bidens pilosa* L. (Asterales: Asteraceae), *Vigna unguiculata* (L.) Walp. (Fabales: Fabaceae), *Capsicum annuum* L. (Solanales: Solanaceae), and *Solanum melongena* L. (Solanales: Solanaceae) were collected from the field of Chinese Academy of Tropical Agricultural Sciences in Haikou, Hainan Province, China (19°58′ N, 110°19′ E).

### 2.2. Behavioral Bioassay

A Y-tube olfactometer, as described by Takabayashi and Dicke [25], was used to examine the behavioral responses of the flower bugs to the flowers of *C. argentea*, *B. pilosa*, *V. unguiculata*, *C. annuum*, and *S. melongena*. These plant species were chosen based on previous field observations of *O. maxidentex* occurring on them in Hainan Island. The olfactometer consisted of a transparent glass tube with a stem of 10 cm in length, a stem angle of 90°, arms of 15 cm in length, and an inner diameter of 1.5 cm. The base of the olfactometer was connected to a tube for releasing the test insects, and the two arms of the glass tube were connected separately to the odor source containing excised plant tissues and the clean air control, respectively. Prior to the assay, excised plant tissues were wrapped with moistened cotton wool around the cut surface and then placed into 200 mL odor source bottles as odor sources. Each part of the olfactometer assembly was connected using colorless, odorless silicone tubing. The sequence of connections was as follows: electric air pump, activated carbon filter, gas-washing cylinders, odor source bottle, atmospheric flow meter, and Y-tube. The airflow in both arms of the Y-tube was regulated at 200 mL/min using the atmospheric flow meter. Air circulation in the room was maintained throughout the assay at a controlled temperature of 26 ± 1 °C and 75 ± 5% RH. Additionally, the Y-tube was covered with a double-layer black cloth to eliminate the potential effects of light on the behavior of the test insects.

The behavioral assay commenced by introducing a single female *O. maxidentex* at the base of the Y-tube main stem. The behavioral responses of the insects were recorded manually in real time by the observer using a stopwatch. Each individual was allowed 5 min to make a choice between the two olfactometer arms. The choice was recorded only when the individual moved beyond two-thirds of an arm and remained there continuously for at least 10 s. Each individual was tested only once. After every 10 individuals, the positions of the two arms were swapped left and right to avoid positional bias. After every 20 individuals, the Y-tube was replaced with a clean one, and the used Y-tube was washed with ethanol solution and dried in an oven at 80 °C before reuse. After every 50 individuals, the plant material in the odor source bottle was renewed. The treatment setup was as follows: the control arm received fresh air, whereas the treatment arm was supplied with flowers of *C. argentea*, *B. pilosa*, *V. unguiculata*, *C. annuum*, or *S. melongena*. Expected values were derived from the null hypothesis that insects exhibit no olfactory preference, yielding a theoretical 1:1 distribution between the two arms. Only insects that made a clear choice were included in observed values, and individuals that remained in the base of the stem arm and failed to make a choice within the allotted time were excluded from the statistical analysis. Each treatment was tested with a total of 100 female individuals, with each responding insect regarded as an independent biological replicate.

### 2.3. Volatile Collection and Identification

Solid-phase microextraction (SPME) was used to collect the volatiles from uninfested *C. argentea* flowers. A total of 2 g of flowers was collected and placed into a 20 mL glass sample vial. The sample volume was limited to no more than two-thirds of the vial height, after which the vial was sealed. SPME fibers were thermally conditioned at 250 °C in the injection port of GC for 50 min prior to use. SPME extraction was performed at 40 °C for 50 min. The fiber was then withdrawn from the sample vial and inserted into the GC injection port, where analytes were thermally desorbed for 6 min.

Gas chromatography–mass spectrometry (GC-MS) was used to identify the volatile compounds collected by SPME. The GC conditions were set as follows: separation was performed on an HP-5MS fused-silica capillary column (30 m × 0.25 mm i.d., 0.25 μm film thickness); carrier gas was high-purity helium at a constant flow rate of 2 mL/min. The inlet temperature was maintained at 250 °C. The GC oven temperature program was set as follows: initial temperature 40 °C, held for 4 min, increased to 160 °C at 5 °C/min and held for 5 min, then further increased to 260 °C at 10 °C/min and held for 5 min. The MS conditions were as follows: solvent delay 2.5 min, transfer line temperature 280 °C, and electron impact (EI) ion source operated at 280 °C. The mass spectrometer was run in EI mode at 70 eV. Data were acquired in full-scan mode over a mass range of 45–550 amu. Volatile collection and GC-MS analysis were performed with three independent biological replicates.

Compound identification was achieved by matching experimental mass spectra with entries in the NIST mass spectrometry (MS) database, in conjunction with chromatographic retention indices. To confirm volatile identity, mass spectra and retention times were compared to those of authentic standards analyzed under identical GC-MS conditions. To control for background contamination, empty 20 mL glass vials were subjected to the exact same SPME extraction and GC-MS procedures as blank controls. Compounds detected in the blank controls, together with fiber-derived impurities such as siloxanes, were considered background noise and excluded from the final floral volatile profile. Finally, only volatile constituents that achieved a matching threshold of 80% in the NIST database and presented in three independent biological replicates were retained for further analysis. Relative abundances of the various volatile compounds were then calculated using peak-area normalization methods based on the total ion chromatograms (TIC).

### 2.4. Electroantennography Responses Assay

EAG was performed to determine the antennal sensitivity of flower bugs to volatile compounds. Each tested compound was diluted in hexane for EAG analysis with the concentrations of 0.01, 0.1, 1, 10, and 100 μg/μL, respectively. The EAG system and protocol used in this study were adapted from previously described methods [26,27]. Briefly, the reference and recording electrodes consisted of two silver wires, each inserted into a glass capillary tube filled with saline solution. Notably, the reference electrode was connected to the excised head rather than the antennal base of *O. maxidentex*, because the antennae were extremely small and rapidly lost sensitivity following excision. The antennal tips were removed to ensure good electrical contact, and the glass capillary of the recording electrode was attached to the distal antennal segment. Test compounds were dissolved in hexane. For each concentration, a 15 μL aliquot of the diluted solution was pipetted onto a Whatman filter paper strip (5 mm × 25 mm). The strip was then inserted into a glass Pasteur pipette to serve as a stimulus cartridge. Filter paper strips loaded with 15 μL hexane alone were prepared in parallel as controls. The Pasteur pipet was inserted into a stainless-steel tube (12 mm in diameter, 200 mm in length) positioned 5 mm away from the antennal preparation. Because antennal responses attenuate over time and vary among individuals, each antenna was stimulated with a hexane control before and after the concentration series (noted as the pre-control CK1 and post-control CK2, respectively).

To evaluate the dose–response relationship, a single successfully mounted antenna was stimulated sequentially with increasing concentrations (from 0.01 to 100 μg/μL) of the specific compound. Stimuli were delivered into a continuous, humidified air stream via an air stimulation controller as a 0.5 s pulse. Signal recording commenced 1 s before the onset of the stimulus pulse and continued for 10 s. To prevent adaptation caused by continuous antennal stimulation, the interval between consecutive stimuli was at least 60 s. The absolute response value of each compound was corrected by subtracting the mean value of the two respective controls (CK1 and CK2) to obtain the normalized EAG response. EAG signals were acquired using a combinatorial probe (IDAC2, Syntech, Kirchzarten, Germany) and analyzed with EAGPro 2 software [28]. Each compound was tested on 10 different female antennae, representing 10 independent biological replicates.

### 2.5. Olfactory Responses of O. maxidentex to Volatile Compounds

Filter paper was cut into 3 cm diameter circles and placed in two odor source bottles. Using a micropipette, a 100 μL aliquot of the test compound solution was applied evenly across the surface of the filter paper in the treatment bottle, while 100 μL of liquid paraffin was applied identically to the filter paper in the control bottle. Each tested compound was diluted in liquid paraffin to prepare serial concentrations of 0.01, 0.1, 1, 10, and 100 μg/μL for olfactory bioassays. Prior to evaluating volatile compounds, a pre-test comparing liquid paraffin (solvent control) against a clean air control was conducted to verify that the solvent itself had no behavioral effect on *O. maxidentex*. This pre-test followed the identical Y-tube olfactometer procedure described previously and utilized a total of 60 female individuals. For the main behavioral bioassays, the compounds tested were consistent with those used in the EAG assay. The remaining experimental procedures were the same as those described for the preceding behavioral bioassays. Each compound treatment was tested with a total of 60 female individuals, with each responding insect regarded as an independent biological replicate.

### 2.6. Chemicals

The 95% 1,3-diethenylbenzene, 98% trans-cinnamaldehyde, 99% methyl salicylate, 98% 3-ethylbenzaldehyde, and 96% 1-nonanal used in the study were purchased from Rhawn Chemistry Technology Co., Ltd. (Shanghai, China). The 97% β-bisabolene was purchased from Bide Pharmatech Ltd. (Shanghai, China). Hexane was purchased from Tianjin Kermel Chemical Reagent Co., Ltd. (Tianjin, China). Paraffin oil was purchased from Shanghai Macklin Biochemical Co., Ltd. (Shanghai, China).

### 2.7. Screening of the Most Attractive Single Compound and Blend

Y-tube olfactometer assays were conducted using the same apparatus and environmental conditions as detailed in Section 2.2. Each treatment was tested with a total of 60 female individuals, with each responding insect serving as an independent biological replicate. Four behaviorally active compounds identified from the olfactory response results were selected for further testing: 1,3-diethenylbenzene, trans-cinnamaldehyde, β-bisabolene, and methyl salicylate. The optimal attractive concentration for each of these four compounds was determined based on the results of the preceding dose–response Y-tube assays (detailed in Section 2.5). These optimal concentrations were then used to generate pairwise combinations in a two-choice bioassay, aimed at identifying the single most attractive compound among the four behaviorally active semiochemicals.

Two distinct synthetic blends were prepared to evaluate their attractiveness. Blend I (natural ratio) was formulated to mimic the natural relative abundance of the four behaviorally active compounds, as determined by GC-MS peak-area normalization. To prepare this blend, stock solutions of 1,3-diethenylbenzene, trans-cinnamaldehyde, β-bisabolene, and methyl salicylate were each prepared at a concentration of 100 µg/μL, and then mixed according to their respective natural proportions detailed in Table S1. Blend II (optimal concentration mixture) was created by combining the four active compounds, with each component added at its previously determined optimal attractive concentration. Specifically, stock solutions of 1,3-diethenylbenzene (100 µg/μL), trans-cinnamaldehyde (0.01 µg/μL), β-bisabolene (10 µg/μL), and methyl salicylate (10 µg/μL) were mixed together in an equal volume ratio (1:1:1:1) (Table S1). A series of Y-tube olfactometer bioassays were then performed to identify the most attractive formulation. First, the behavioral responses of *O. maxidentex* to blend I and blend II were individually tested against a solvent control (CK) to confirm their attractiveness. Next, a direct pairwise comparison between blend I and blend II was conducted to determine the more potent mixture. Finally, the more attractive blend was directly compared with the single most attractive compound, β-bisabolene (tested at its optimal concentration of 10 μg/μL). For each of these pairwise comparisons, behavioral responses were recorded with 60 female individuals (n = 60), following the same procedure as described above.

### 2.8. Statistical Analysis

All statistical analyses were performed using SPSS 25.0 (SPSS, Inc., Chicago, IL, USA), and figures were generated with GraphPad Prism 8 (GraphPad Software, LLC., Boston, MA, USA). For all Y-tube olfactometer bioassays, a Chi-square test (χ^2^) was used to determine the significance of behavioral choices between the two arms. The assumptions for this test were satisfied in all cases, as the expected frequency for each arm was well above the minimum threshold of 5 (e.g., 50 for an experiment with n = 100 responding individuals). For the chemical analysis, the relative abundance of the identified volatiles was calculated using peak-area normalization based on the total ion chromatograms (TIC). No internal standard was used for quantification, and thus, the data represent relative proportions rather than absolute concentrations. For the EAG data, the significance of EAG amplitudes between across compound concentrations was determined by one-way analysis of variance (One-way ANOVA). Prior to analysis, the data were tested for normality (Shapiro–Wilk test) and homogeneity of variance (Levene’s test). Since these assumptions were met, ANOVA was followed by Tukey’s HSD post hoc test for multiple comparisons (*p* < 0.05).

## 3. Results

### 3.1. Behavioral Responses of O. maxidentex to Plant Odors

**Figure 2:**
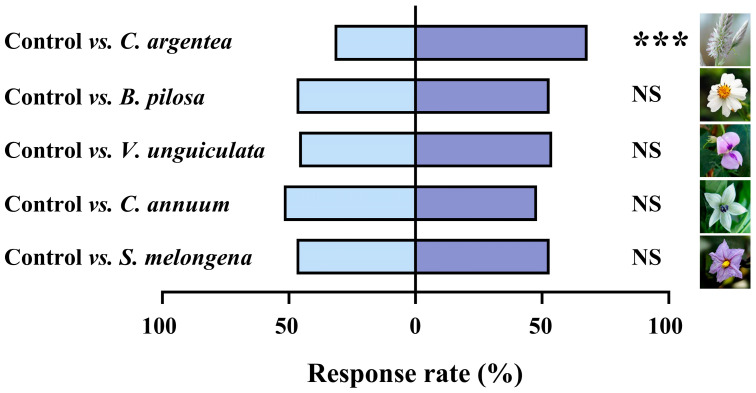
Behavioral responses of female *Orius maxidentex* Ghauri to different plants. One hundred female individuals were tested for each treatment. Asterisks indicate statistically significant differences (*** *p* < 0.001), whereas NS indicates no significant difference (*p* > 0.05).

In Y-tube olfactory bioassays, the results showed that female *O. maxidentex* were significantly attracted to the volatiles emitted by *C. argentea* (χ^2^ = 12.960, df = 1, *p* < 0.001) compared with the control (Figure 2). There was no significant difference between the other plants and the control (*B. pilosa*: χ^2^ = 0.360, df = 1, *p* = 0.549; *V. unguiculata*: χ^2^ = 0.640, df = 1, *p* = 0.424; *C. annuum*: χ^2^ = 0.160, df = 1, *p* = 0.689; *S. melongena*: χ^2^ = 0.360, df = 1, *p* = 0.549).

### 3.2. Volatile Identification from the Flowers of C. argentea

**Figure 3:**
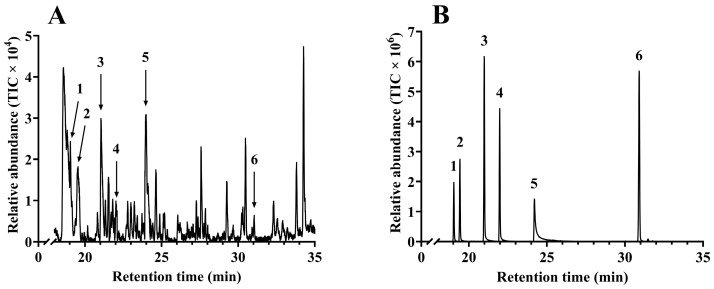
Total ion chromatograms (TIC) for the extracts of *Celosia argentea* L. and authentic standards. (**A**) *C. argentea*. (**B**) Authentic standards. Peaks of compounds were identified as 1: nonanal; 2: 1,3-diethenylbenzene; 3: 3-ethylbenzaldehyde, 4: methyl salicylate, 5: trans-cinnamaldehyde, 6: β-bisabolene.

The floral volatiles from *C. argentea* samples were extracted by SPME and analyzed by GC-MS. Six compounds were identified, including nonanal, 1,3-diethenylbenzene, 3-ethylbenzaldehyde, methyl salicylate, trans-cinnamaldehyde, and β-bisabolene, and their retention times are presented in Table 1. Chromatograms obtained by GC-MS analysis are shown in Figure 3A, and these compounds were confirmed by comparing their retention times and fragmentation patterns with those of authentic standards (Figure 3B).

### 3.3. EAG Responses of O. maxidentex Antennae to Volatile Compounds

**Figure 4:**
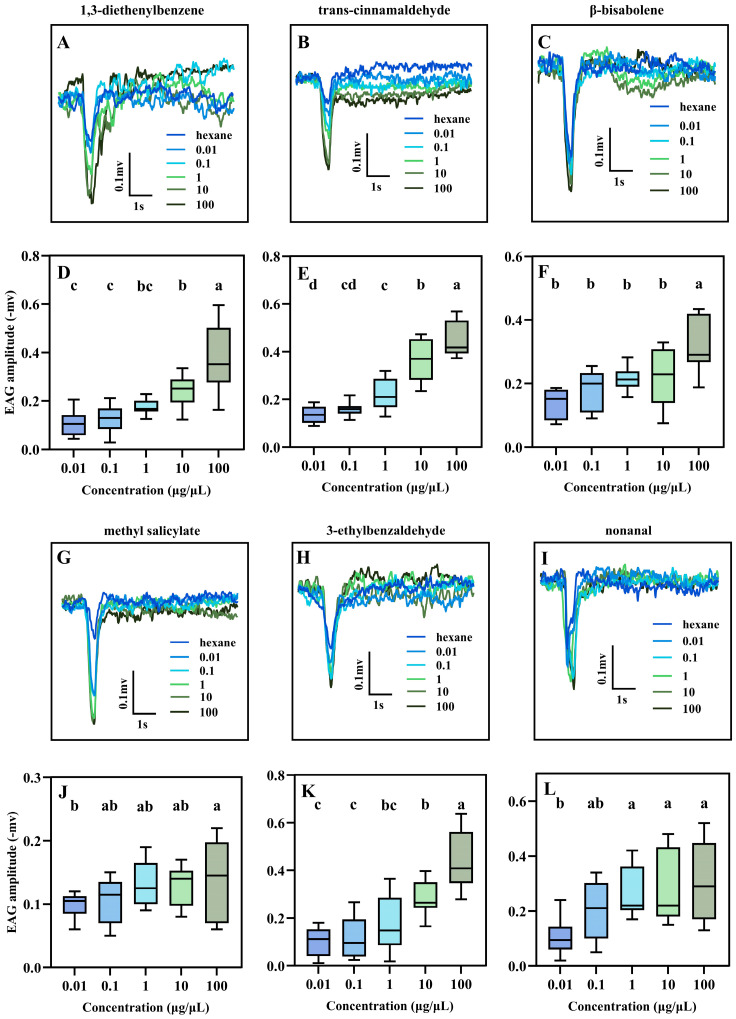
EAG responses of *Orius maxidentex* Ghauri induced by *Celosia argentea* L. volatile compounds. (**A**–**C**, **G**–**I**) representative EAG trace of each volatile compound, including 1,3-diethenylbenzene, trans-cinnamaldehyde, β-bisabolene, methyl salicylate, 3-ethylbenzaldehyde, and nonanal. (**D**–**F**, **J**–**L**) EAG amplitude stimulated by each volatile compound at different concentrations. Bars with different letters indicate statistically significant differences between concentrations (*p* < 0.05).

All six tested compounds elicited significant, dose-dependent EAG responses from the antennae of *O. maxidentex* (Figure 4). For β-bisabolene, a clear dose–response relationship was observed, with the EAG amplitude at 100 µg/µL being significantly higher than those at all other lower concentrations (F = 10.140, df = 4, *p* < 0.001). A similar pattern of increasing response with concentration was observed for 3-ethylbenzaldehyde (F = 23.761, df = 4, *p* < 0.001) and 1,3-diethenylbenzene (F = 19.437, df = 4, *p* < 0.001). For both compounds, the responses at 100 µg/µL were significantly higher than those at 10 µg/µL and all lower concentrations. Trans-cinnamaldehyde elicited a particularly strong dose-dependent response, with significant differences detected between most of the tested concentrations (F = 48.519, df = 4, *p* < 0.001). Methyl salicylate triggered the highest EAG amplitude at 100 μg/μL in *O. maxidentex*, and the EAG amplitudes at 0.01, 0.1, 1, and 10 μg/μL showed no significant difference (F = 2.163, df = 4, *p* = 0.089). For nonanal, the EAG amplitude at 0.1, 1, 10 and 100 μg/μL showed no significant difference but were significantly different from that at 0.01 μg/μL (F = 5.227, df = 4, *p* = 0.002). These results demonstrate that all six compounds are electrophysiologically active, indicating that the peripheral olfactory system of *O. maxidentex* is capable of detecting them.

### 3.4. Behavioral Responses of O. maxidentex to Volatile Compounds

**Figure 5:**
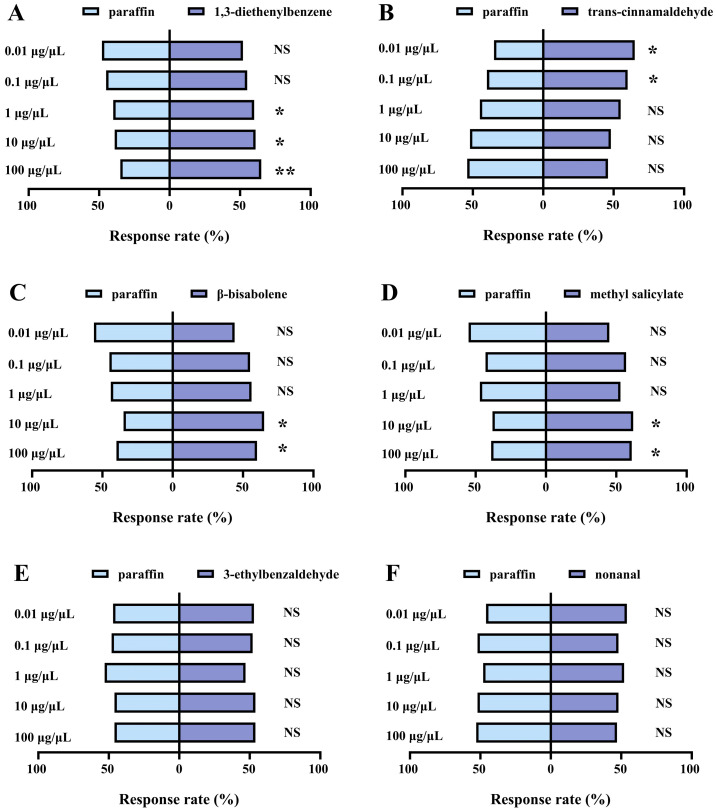
Behavioral responses of *Orius maxidentex* Ghauri induced by *Celosia argentea* L. volatile compounds at different concentrations. (**A**) 1,3-diethenylbenzene; (**B**) trans-cinnamaldehyde; (**C**) β-bisabolene; (**D**) methyl salicylate; (**E**) 3-ethylbenzaldehyde; (**F**) nonanal. Sixty female individuals were tested in each treatment. Asterisks indicate statistically significant differences between concentrations (* *p* < 0.05, ** *p* < 0.01), whereas NS indicates no significant difference (*p* > 0.05).

In the Y-tube olfactory assays, 1,3-diethenylbenzene significantly attracted *O. maxidentex* at the concentrations of 1 μg/μL (χ^2^ = 4.000, df = 1, *p* = 0.046), 10 μg/μL (χ^2^ = 4.840, df = 1, *p* = 0.028), and 100 μg/μL (χ^2^ = 9.000, df = 1, *p* = 0.003). However, *O. maxidentex* showed no preference for 1,3-diethenylbenzene at 0.01 μg/μL (χ^2^ = 0.160, df = 1, *p* = 0.689) and 0.1 μg/μL (χ^2^ = 1.000, df = 1, *p* = 0.317) (Figure 5A). Trans-cinnamaldehyde significantly attracted *O. maxidentex* at the concentrations of 0.01 μg/μL (χ^2^ = 9.000, df = 1, *p* = 0.003) and 0.1 μg/μL (χ^2^ = 4.000, df = 1, *p* = 0.046). However, *O. maxidentex* showed no preference for trans-cinnamaldehyde at 1 μg/μL (χ^2^ = 1.000, df = 1, *p* = 0.317), 10 μg/μL (χ^2^ = 0.160, df = 1, *p* = 0.689), and 100 μg/μL (χ^2^ = 0.640, df = 1, *p* = 0.424) (Figure 5B). β-bisabolene significantly attracted *O. maxidentex* at the concentrations of 10 μg/μL (χ^2^ = 9.000, df = 1, *p* = 0.003) and 100 μg/μL (χ^2^ = 4.000, df = 1, *p* = 0.046). However, *O. maxidentex* showed no preference for β-bisabolene at 0.01 μg/μL (χ^2^ = 1.440, df = 1, *p* = 0.230), 0.1 μg/μL (χ^2^ = 1.000, df = 1, *p* = 0.317), and 1 μg/μL (χ^2^ = 1.440, df = 1, *p* = 0.230) (Figure 5C). Methyl salicylate significantly attracted *O. maxidentex* at the concentrations of 10 μg/μL (χ^2^ = 5.760, df = 1, *p* = 0.016) and 100 μg/μL (χ^2^ = 4.840, df = 1, *p* = 0.028). However, *O. maxidentex* showed no preference for methyl salicylate at 0.01 μg/μL (χ^2^ = 1.440, df = 1, *p* = 0.230), 0.1 μg/μL (χ^2^ = 1.960, df = 1, *p* = 0.162), and 1 μg/μL (χ^2^ = 0.360, df = 1, *p* = 0.549) (Figure 5D). *Orius maxidentex* showed no preference for 3-ethylbenzaldehyde (0.01 μg/μL: χ^2^ = 0.360, df = 1, *p* = 0.549; 0.1 μg/μL: χ^2^ = 0.160, df = 1, *p* = 0.689; 1 μg/μL: χ^2^ = 0.360, df = 1, *p* = 0.549; 10 μg/μL: χ^2^ = 0.640, df = 1, *p* = 0.424; 100 μg/μL: χ^2^ = 0.640, df = 1, *p* = 0.424) and nonanal (0.01 μg/μL: χ^2^ = 0.640, df = 1, *p* = 0.424; 0.1 μg/μL: χ^2^ = 0.160, df = 1, *p* = 0.689; 1 μg/μL: χ^2^ = 0.160, df = 1, *p* = 0.689; 10 μg/μL: χ^2^ = 0.160, df = 1, *p* = 0.689; 100 μg/μL: χ^2^ = 0.640, df = 1, *p* = 0.424) at all concentrations (Figure 5E,F).

### 3.5. Screening of the Most Attractive Single Compound and Blend to O. maxidentex

**Figure 6:**
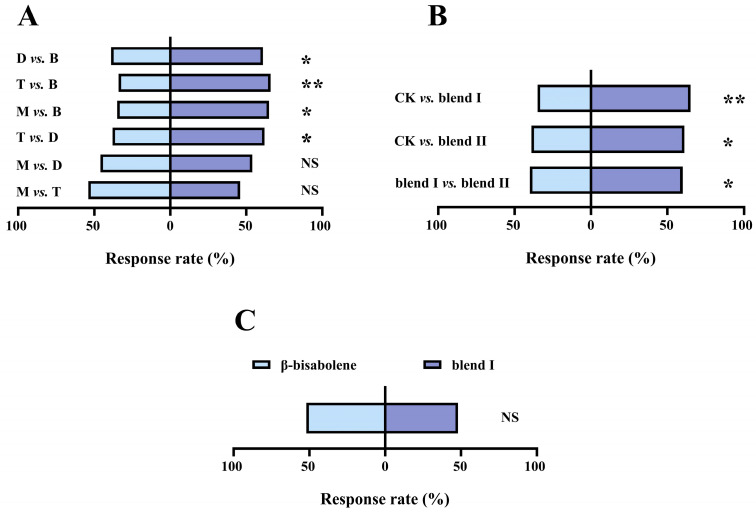
Screening of the most attractive single compound and blend to *Orius maxidentex* Ghauri. (**A**) Behavioral responses of *O. maxidentex* to optimal concentrations of four active compounds. “B” stands for 10 μg/μL β-bisabolene, “D” stands for 100 μg/μL 1,3-diethenylbenzene, “M” stands for 10 μg/μL methyl salicylate, and “T” stands for 0.01 μg/μL trans-cinnamaldehyde; (**B**) behavioral responses of *O. maxidentex* to blend I and II; (**C**) behavioral responses of *O. maxidentex* to 10 μg/μL β-bisabolene and blend I. Sixty female individuals were tested in each treatment. Asterisks indicate statistically significant differences (* *p* < 0.05, ** *p* < 0.01), whereas NS indicates no significant difference (*p* > 0.05).

At a concentration of 10 μg/μL, β-bisabolene significantly attracted *O. maxidentex* compared with 1,3-diethenylbenzene (χ^2^ = 4.840, df = 1, *p* = 0.028), trans-cinnamaldehyde (χ^2^ = 10.240, df = 1, *p* = 0.001), and methyl salicylate (χ^2^ = 9.000, df = 1, *p* = 0.003). At a concentration of 100 μg/μL, 1,3-diethenylbenzene significantly attracted *O. maxidentex* compared with trans-cinnamaldehyde (χ^2^ = 5.760, df = 1, *p* = 0.016). No significant differences were observed between other treatments (methyl salicylate vs. 1,3-diethenylbenzene: χ^2^ = 0.640, df = 1, *p* = 0.424; methyl salicylate vs. trans-cinnamaldehyde: χ^2^ = 0.640, df = 1, *p* = 0.424) (Figure 6A). Thus, 10 μg/μL β-bisabolene was the most attractive single volatile. Compared with the control, blend I (χ^2^ = 9.000, df = 1, *p* = 0.003) and blend II (χ^2^ = 4.840, df = 1, *p* = 0.028) significantly attracted *O. maxidentex*. Blend I significantly attracted *O. maxidentex* compared with blend II (χ^2^ = 4.000, df = 1, *p* = 0.046) (Figure 6B). Finally, *O. maxidentex* showed no significant preference between blend I and β-bisabolene at 10 μg/μL (χ^2^ = 0.160, df = 1, *p* = 0.689) (Figure 6C).

## 4. Discussion

In the present research, we investigated the chemosensory cues underlying the floral habitat selection of the flower bug. Specifically, we tested whether this generalist predator is able to identify the major compounds released in the constitutive floral volatiles of its host plant *C. argentea*, in the absence of HIPVs. Our results show that the predatory bug effectively responds to the floral volatiles of its host. On the one hand, this chemically mediated orientation to flowering plants likely reflects an adaptive strategy to locate a microhabitat, which provides them with shelter, alternative food, and oviposition sites [24]. It is known that females, during host selection for oviposition to ensure that the plant provides the nutritional needs and best development of offspring [29,30], a principle known as “mother knows best” or preference-performance hypotheses [31], allowing the newly hatched *O. maxidentex* nymphs to feed on supplementary plant foods at early instar stages. Moreover, the mature inflorescences of *C. argentea* also attract thrips pests, such as *Frankliniella intonsa* (Trybom) (Thysanoptera: Thripidae) [32], creating a prey-rich niche for *O. maxidentex* and allowing senior instar stages to forage efficiently. On the other hand, the recruitment of predators via VOCs represents a mutualistic interaction: the plant attracts predators to control pests, thereby reducing herbivore damage and potentially enhancing fitness [33].

The fragrance signature of flowers is a composite of volatile chemicals in specific stochiometric concentrations [34]. Typically, these compounds are monoterpenoids, sesquiterpenoids, benzenoids, phenylpropanoids, and fatty acid derivatives [29]. Numerous studies have shown that insects respond to complex mixtures of plant VOCs, yet these blends can often be simplified to a smaller number of key components while retaining their functional attractiveness [35]. In this study, 1,3-diethenylbenzene, trans-cinnamaldehyde, β-bisabolene, methyl salicylate, 3-ethylbenzaldehyde and nonanal produced significant antennal responses from *O. maxidentex* in the EAG analyses, indicating that the peripheral olfactory system of the predator is tuned to recognize these semiochemicals. Among them, behavioral attraction was found for 1,3-diethenylbenzene, trans-cinnamaldehyde, β-bisabolene, and methyl salicylate, suggesting that these may be the key compounds mediating floral recognition. Further, the most attractive single compound was identified as 10 μg/μL β-bisabolene. To our knowledge, this is the first study to report antennal sensitivity to these six semiochemicals in *O. maxidentex*, although several of them are known to elicit antennal depolarizations or behavioral responses in other insects. Likewise, trans-cinnamaldehyde, emitted by *Cytinus* (Malvales: Cytinaceae) inflorescences, elicits electrophysiological and behavioral responses in the ant *Aphaenogaster senilis* Mayr (Hymenoptera: Formicidae) [2]. Trans-cinnamaldehyde also exerted strong fumigant and contact toxicity against house dust mites *Dermatophagoides farinae* Hughes and *Dermatophagoides pteronyssinus* (Trouessart) (Sarcoptiformes: Pyroglyphidae) [36], booklice *Liposcelis bostrychophila* Badonnel (Psocodea: Liposcelididae) [37], cigarette beetle *Lasioderma serricorne* (F.) (Coleoptera: Ptinidae) [38] and adzukii bean weevil *Callosobruchus chinensis* L. (Coleoptera: Chrysomelidae) [39]. Furthermore, it also elicited toxicity toward the spotted-wing drosophila *Drosophila suzukii* Matsumura (Diptera: Drosophilidae) adults and eggs [40]. The ecological role of β-bisabolene appears to be highly species- and context-dependent. For instance, while it exhibits repellency against *Aedes albopictus* (Skuse) (Diptera: Culicidae) [41], in wild potato accessions it repelled aphids *Myzus persicae* (Sulzer) (Hemiptera: Aphididae) and attracted their natural enemy *Diaeretiella rapae* (McIntosh) (Hymenoptera: Braconidae) [42]. Our finding that it acts as a key attractant for *O. maxidentex* further underscores this functional diversity. As a typical semiochemical cue often associated with HIPVs, methyl salicylate is a well-known attractant for numerous natural enemy species, such as *Neoseiulus californicus* (McGregor) (Acari: Phytoseiidae) [43], *Chrysopa nigricornis* Burmeister (Neuroptera: Chrysopidae) [44], *Stethorus punctum* (LeConte) (Coleoptera: Coccinellidae), and *Geocoris pallens* Stal (Hemiptera: Geocoridae) [45]. In addition, several studies have shown that methyl salicylate can act as a repellent for several aphid species when they leave their overwintering places to colonize host plants in spring [46,47]. Although we identified it as a constitutive floral volatile in an uninfested plant, its presence suggests a potential pre-emptive defensive strategy by the plant, warranting further investigation into its role in mediating multitrophic interactions under field conditions. Similarly, while nonanal is a common plant volatile known to attract predators like *Harmonia axyridis* (Pallas) (Coleoptera: Coccinellidae) [48,49], it did not elicit a significant behavioral orientation in *O. maxidentex* in our bioassays. This finding highlights the crucial distinction between mere antennal detection (an EAG response) and actual behavioral attraction, underscoring the species-specific nature of olfactory selectivity.

Although the attraction of single volatile compounds to insects has been extensively reported, the ratio of volatiles emitted by the plant is an even more vital component of the olfactory signal coding [50]. Two hypotheses have been proposed on how insects recognize their host plants [51]. Hypothesis I proposes that specific compounds are used for host detection by insects, which is the “token stimulus” theory proposed by Fraenkel [52]. Hypothesis II proposes that plant odor specificity is achieved by a particular ratio between constituent volatiles distributed generally among plant species. It has been suggested that the olfactory systems of contemporary insects originated ~500 million years ago in the Paleozoic era, and that plants and insects have been co-evolving ever since [53]. Over time, it is not the case that specialist and generalist insects have evolved different olfactory receptor neurons (ORNs), indicating hypothesis II is generally accepted [50]. Hence, we conducted experiments on the behavioral responses of *O. maxidentex* to different formulations of blends. Blend I, which mimicked the natural ratio of constituent compounds, elicited more significant attraction than blend II, which was composed of the optimal active concentration of each compound. The result indicates that *O. maxidentex* is ecologically adapted to plant volatiles in specific ratios as naturally produced. This is consistent with findings in honey bees *Apis mellifera* Linnaeus (Hymenoptera: Apidae), which were also highly responsive to the flower mixtures reconstructed at natural ratios [54]. Notably, blend I was as effective as β-bisabolene, the most attractive single compound, in the Y-tube olfactory assays. This suggests that while the full blend provides a complete recognition signal, β-bisabolene serves as a dominant, perhaps primary, cue driving the orientation behavior. However, the strong effect of this single compound does not negate the importance of the full blend. Rather, it is likely that both the presence of a key attractant like β-bisabolene and the specific ratio of the entire volatile mixture act synergistically to create a robust and reliable signal for habitat selection.

In summary, the results demonstrated that the floral volatiles of uninfested *C. argentea* play a key role in the habitat selection and orientation behavior of *O. maxidentex*. We also revealed that diverse semiochemicals extracted from flowers are involved in floral volatile compound composition, and identified β-bisabolene as a critical attractant through electrophysiological and behavioral responses of *O. maxidentex*. However, it is crucial to emphasize that these conclusions are based on controlled laboratory assays, and their translation to field conditions requires careful validation. Much is still to be evaluated before proposing a protocol for using these VOCs in fields: (i) further studies should evaluate whether these semiochemicals influence target pests in the field while exerting an attractive effect on predatory bugs; (ii) field experiments combining synthetic semiochemicals with oviposition substrates could be conducted to evaluate their effect on the attraction and retention of predatory bugs. Addressing these points will contribute to a more comprehensive understanding of the potential application of VOCs for predatory bugs in field pest control. Furthermore, understanding the behavioral manipulation of predatory bugs using semiochemicals provides valuable insights into the “push-pull” strategy and conservation biological control, thereby reducing reliance on external interventions such as insecticide applications. Our results provide insights into the chemical ecology of the flower bug *O. maxidentex* and may have practical utility for sustainable pest management.

## 5. Conclusions

In this study, we elucidated the chemosensory cues underlying the habitat selection and host preference of the generalist predator *O. maxidentex*. Our results demonstrate that the strong attraction of *O. maxidentex* to the flowers of *C. argentea* is mediated by a specific blend rather than random foraging. We identified six volatile compounds, among which β-bisabolene was determined to be the key semiochemical, eliciting both significant EAG responses and strong behavioral attraction comparable to the natural volatile blend. These findings confirm that *O. maxidentex* has evolved specific olfactory adaptations to locate its floral niche, allowing it to utilize *C. argentea* as a refuge and food source even in the absence of prey. While these laboratory-based findings require further field validation, they provide a theoretical basis for utilizing *C. argentea* as a banker plant or β-bisabolene-based attractants to enhance *O. maxidentex* populations in biological control.

## Figures and Tables

**Table 1:** Volatile compounds emitted by *Celosia argentea* L.

Number	Retention Time (Min)	Compound	CAS Number	Peak Area (%)
1	19.050	nonanal	124-19-6	4.27 ± 1.04
2	19.543	1,3-diethenylbenzene	108-57-6	10.34 ± 1.95
3	21.052	3-ethylbenzaldehyde	34246-54-3	6.52 ± 1.35
4	22.017	methyl salicylate	119-36-8	7.26 ± 1.12
5	24.031	trans-cinnamaldehyde	14371-10-9	9.53 ± 0.74
6	31.049	β-bisabolene	495-61-4	7.76 ± 0.96

Values are presented as mean ± standard deviation (SD) of three independent biological replicates (n = 3).

## Data Availability

Data are available on request.

## Supplementary Materials

The following supporting information can be downloaded at: https://www.mdpi.com/article/10.3390/biology15080658/s1, Table S1: Formulation of blend I and II.
