# Supplementary material for: Floral Niche Selection by a Generalist Predator: Chemo-Orientation of Orius maxidentex to Celosia argentea Volatiles
Source: Biology (Basel). 2026 Apr 21;15(8):658. doi: 10.3390/biology15080658 (PMC13113461; doi:10.3390/biology15080658)
Supplement: Supplementary file 1 [file biology-15-00658-s001.zip › Table_S1.pdf]

Table S1. Formulation of blend I and blend II.

Type	Compound	Concentration	Proportion	Solvent
blend I	1,3-diethenylbenzene	100 μg/μL	1034	liquid paraffin
	trans-cinnamaldehyde	100 μg/μL	953	liquid paraffin
	β-bisabolene	100 μg/μL	776	liquid paraffin
	methyl salicylate	100 μg/μL	726	liquid paraffin
blend II	1,3-diethenylbenzene	100 μg/μL	1	liquid paraffin
	trans-cinnamaldehyde	0.01 μg/μL	1	liquid paraffin
	β-bisabolene	10 μg/μL	1	liquid paraffin
	methyl salicylate	10 μg/μL	1	liquid paraffin
